# Pediatric oral health: community-based participatory research

**DOI:** 10.1186/s12887-022-03153-0

**Published:** 2022-02-15

**Authors:** Marcella Ogenchuk, Juanita Graham, Gerry Uswak, Holly Graham, Robert Weiler, Vivian R. Ramsden

**Affiliations:** 1 College of Nursing, University of Saskatchewan, HSc E Wing 4226, 104 Clinic Place, Saskatoon, Saskatchewan S7N 5E5 Canada; 2 Thunderchild First Nation, Box 744, Turtleford, Saskatchewan S0M 2Y0 Canada; 3 College of Dentistry, University of Saskatchewan, Saskatoon, Room 303 DC, 105 Wiggins Road Sk. S7N 5E4 Canada; 4 College of Nursing, University of Saskatchewan, HSc E Wing 4224, 104 Clinic Place, Saskatoon, Saskatchewan S7N 5E5 Canada; 5 Department of Anaesthesia, University of Saskatchewan, Saskatoon, Canada; 6 Research Division, Department of Academic Family Medicine, University of Saskatchewan, WWPHC, 3311 Fairlight Drive, Saskatoon, SK S7M 3Y5 Canada

**Keywords:** Pediatric oral health, Indigenous, Community-based participatory research, Transformation

## Abstract

**Background:**

The most common chronic disease affecting children in Canada is dental caries. The objective of this study was to explore, identify, and address the strengths and barriers related to oral health services with an independent Indigenous community in Saskatchewan.

**Methods:**

Community-based participatory research used interviews with Elders, health care providers, teachers, and parents/guardians of elementary school-aged children. The research focused on the development of genuine partnerships with the community. During data collection, the findings/results were returned to the community to establish direction, build success, and establish next steps. Thematic analysis was undertaken with the community. Descriptive statistics were analyzed using SPSS.

**Results:**

The most commonly identified themes included: community resilience; the need for resource development and process to improve oral health literacy and skills; and how access to care barriers dually affected and related to personal and community cost, time, and human resources.

**Conclusions:**

The research process involved the co-creation of tools to identify strengths within the community and drive opportunities for change; subsequently generating solutions to the practical problems and potentially transform the health system accessed by the community.

## Background

Although dental caries is common in Canadian children today, there is evidence that it was not always as prevalent in Indigenous communities [1–4]. Archeological findings of Inuit people show exceptionally low instances of dental caries compared to present day levels; indicating that Indigenous communities had generational knowledge of oral health and practices [3, 5]. The inequities in health status—including oral health, —affecting Indigenous populations are an impact of colonialism [6, 7]. Traditional oral health care approaches incorporated the use of animal sinew as dental floss, and birch bark as an oral antibacterial treatment [8]. As fermentable carbohydrates rich foods increased in availability, dental caries became widespread [1]. Introduction of new high-sugar food coupled with poor access to dental care has been identified as a contributor to a strong rise in dental caries cases in Canadian Indigenous communities [9, 10].

Oral health impacts general health and quality of life and there is increasing evidence of a direct link between oral and systemic disease(s) [11]. Early childhood caries (ECC) is defined by the presence of decayed or filled tooth surfaces, on one or more missing teeth due to dental caries in a child between birth and 71 months of age [12]. Tooth decay is the most prevalent chronic childhood disease in Canada [13] with Saskatchewan having the third highest rate, after Nunavut and Northwest Territories, of day surgery operations to treat dental caries among children aged 1-5 years [14]. Children who are treated for tooth decay in dentists’ offices or clinics are not included in the data; therefore, underestimating the severity of the problem. ECC can have significant consequences for functional, psychological, and physical wellbeing of children [15]. Additional health problems, such as low birth weight [16], pre-term delivery [17] and iron deficiency [18] have been shown to be linked to ECC. In Canada, 57% of children aged 6-11 years old are infected with ECC, while 24% of all children have damage to their permanent teeth related to caries [19]. Children experiencing ECC can have difficulty eating, and speaking, and suffer pain that can lead to difficulty sleeping [2]. Children and adolescence with caries experience poor school performance and decreases in their self-confidence [2, 19]. ECC also presents a burden to the healthcare system costing $21.2 million annually (inclusive of day surgery) to treat affected patients [14]. Despite the fact that ECC is preventable and when caught early is treatable in the community, advanced decay frequently involves surgical intervention under general anaesthesia, placing these children at an increased health risk [14]. Although the root cause of ECC is a bacterial infection, it is considered a multifactorial disease from a combination of dietary choices, bacteria exposure level, and other social determinants of health [14].

The Canadian Academy of Health Sciences’ (CAHS) Report [20] on the issue of access to oral health care among vulnerable populations in Canada examined inequalities between social groups in Canada. Analysis of the Canadian Health Measures Survey (CHMS) data revealed that vulnerable populations experiencing lower socioeconomic status were less likely to have dental insurance, and therefore more likely to avoid the dentist due to cost except in the case of an emergency [19]. This leads to higher instances of untreated dental decay leading to pain, missing teeth, and gum disease. In some cases, members of the affected populations would also be more likely to avoid eating healthy foods such as fruits and vegetables due to dental pain. Challenges in utilizing oral health care services were identified in the Report and included the five dimensions proposed by Pechansky and Thomas [21]: affordability (do the provider’s charges relate to the client’s ability to pay for services?); availability (does the provider have the requisite resources, such as personnel and technology to meet the needs of the client?); accessibility (how easily can the client physically reach the provider’s location?); accommodation (is the provider’s operation organized in ways that meet the constraints and needs of the client?); and acceptability (is the client comfortable with the characteristics of the provider and vice versa?) [21]. Overall, addressing inequalities is recognized as a critical part of improving oral health in Indigenous communities. This can be more effective when employed in a culturally sensitive framework that addresses concerns particular to Indigenous communities, such as maintaining traditions [22].

The Federal Government funds health transfers to the provinces and territories via the Canada Health Act. For the most part, oral health falls outside of the Canada Health Act; consequently, there is considerable variation in services, continuity of programming and portability of benefits from one province or territory to another [20]. Services are provided through a variety of programs, funding arrangements and oral health care providers in First Nations communities. Despite funding provided, the services that are available are not always accessible to the entire community. Indigenous communities show the highest rates of dental decay compared to non-Indigenous populations; 2-3 times higher in some cases [23]. The initial Canadian Oral Health Strategy (COHS) (2005-2010) was developed without a significant mandate to improve oral health and was lacking in baseline data about the prevalence of ECC to develop programs or monitor changes [23]. There has been one First Nations Oral Health Survey [24] completed and an Inuit Oral Health Survey [25] published since then. A Children’s Oral Health Initiative (COHI) was created to prevent dental caries among pregnant women and young children and parents of First Nations and Inuit ethnicity [26]. COHI utilizes community-based aides to support oral health care professionals in oral health education and oral health services [26]. Findings to date suggest that access to preventive oral health services to children has improved but prevalence of dental caries has not decreased despite the COHI [27]. The foundational planning, however, did result in a process that focused on targeted strategies for a less-advantaged population and priority groups, including Indigenous peoples and low-income families.

This study explored the perceptions of Indigenous community members on the impact of oral health on children in Canada. This included the current practices in implementing health promotion, prevention, and treatment to improve the oral health status of children in an Indigenous community. The study also examined the barriers that parents and the community perceived exist to accessing basic health promotion, prevention, and treatment of oral health resources in children. Based on the services currently available, further implementation of programs that address oral health in meaningful ways were also explored with the community members.

## Methods

### Study area, design and period

The Community: Thunderchild First Nation is an independent Cree First Nation in Saskatchewan, Canada and is located approximately 113 km northwest of North Battleford, SK [28]. This First Nation does not have any affiliation with any Tribal Council. The community has a rich and diverse population of Plains Cree people, and many of the community members are fluent in both Cree and English. This First Nation has a population of 1868 with 630 residing on the reserve [28]. This research aligns with the Tri-Council Policy Statement (TCPS 2) [29] specifically Chapter 9, utilizing participatory health research and transformative action research [30–32]. This project focused on developing relationships with the community and forming authentic partnerships to address the existing oral health issues in a culturally safe way [32]. Community-based participatory research, uses a partnership framework that equitably engages community members and researchers in all aspect of the process [30]. This research encompassed a high level of community participation where the researchers took direction from the community leaders and stakeholders; thus mutual learning and research were undertaken together [30]. The main stakeholders of the results of this study are the community members themselves, as well as the community leaders and organizers. Stakeholder engagement has been recognized as an important tool for informing change based on results/findings that evolve from the research. This study utilized stakeholder engagement principles of organizational factors, values, and practices as outlined by Boaz et al [33] in the use of community-based participatory research by including the administrators in the development of the research focus, the research questions, methods and participant recruitment.

### Approach (stage 1)

The researchers, along with the Thunderchild Advisory Board, formalized the proposal and the Application for Behavioural Ethics Review. Once the content was agreed upon, the proposal was submitted to the University of Saskatchewan’s Behavioural Research Ethics Board for consideration and approval. It was approved on August 22, 2017 (Beh 17-305).

### Engagement (stage 2)

Community leaders helped to identify and invite participants that met the inclusion criteria to participate in the research project [29]. The Thunderchild First Nation Advisory Board, provided recommendations of research participants, such as Elders, health care providers and teachers. The community led the process for recruitment of the Research Assistants and the participants through newsletter and Facebook to participate in semi-structured interviews. Parents/guardians of pre-school or school-aged children were invited to participate through community advertising (Facebook, school newsletters). Informed consent was obtained as outlined in the ethics proposal, from all participants. Semi-structured interviews were conducted with Elders, health care providers, teachers, and parents/guardians of elementary school-aged children. The number of participants invited to participate in this project were chosen to facilitate rich data and saturation [34]. Data collected with Elders and community health workers facilitated validity of the data.

### Data analysis (stages 3 & 4)

Descriptive statistics were analyzed using the Statistical Package for the Social Sciences (SPSS v17) to provide a profile of those that participated in the research project. Each participant was given an opportunity to review the written copy and modify the content following the interview. The interviews were transcribed, and an inductive, thematic analysis undertaken with the community [35]. The data was analyzed by the researchers and research assistants as a form of member-checking. The preliminary findings which included the themes that evolved from the data were presented to a small group; as well as one-on-one with individual stakeholders in the community. The feedback from these meetings assisted in the interpretation of the findings presented in this paper. This step was important to ensure that the analysis was context correct and resonated with the community. Returning to the community to share the results/findings promoted shared knowledge. An individual member who provided in depth feedback is included as an author. This research achieved theoretical sufficiency from the findings; evolving from the semi-structured interviews with community leaders, health care providers, and parents that occurred through the ongoing transcription and data collection process [34].

## Results

### Socio-demographic characteristics

Twenty-eight community members participated in the survey. The majority of participants were female (23/28; 82%) between 40 and 59 years of age (14/28; 50%) (Tables 1 and 2).

**Table 1:** Total participants

Gender	Number of Participants (%)
Male	5 (18)
Female	23 (82)
Total	28

**Table 2:** Participant age

Age in Years	Number of Participants (%)
20-29	4 (14.3)
30-39	5 (17.9)
40-49	8 (28.6)
50-59	6 (21.4)
60-69	3 (10.7)
70-79	1 (3.6)
80-89	1 (3.6)
Total	28

Twenty nine percent of the participants (8/28) said that Cree was their first language and 50% (14/28) of participants responding to the survey said that English was their first language. An additional 21% (6/28) said that they spoke Cree and/or English as their first language (Table 3).

**Table 3:** First language

First Language	Number of Participants (%)
Cree	8 (28.6)
English	14 (50.0)
Cree/English	6 (21.4)
Total	28

Over half of the participants were employed (16/28; 57%) (Table 4).

**Table 4:** Employment of participants

Employed	Number of Participants (%)
Yes	16 (57.1)
No	10 (35.7)
Did Not Answer	2 (7.1)
Total	28

Seventy nine percent (22/28) of the participants said that children lived in the household. Thirty six percent (10/28) of the children were < than 5 years of age and 43% (12/28) were > than 5 years of age. Eighteen percent of participants said that there were older adults or Elders living in the household. Almost half, 53% (15/28) of participants reported that they lived in homes with four or fewer people, and 11 of these 15 (39%) participants lived in homes with only 1-2 people (Tables 5 and 6). Almost half (13/28) of the participants were living in homes with five or more people.

**Table 5:** Home occupants in different age groups

Age Group	Number of Homes (%)
Children under 5 years	10 (35.7)
Children older than 5 years	12 (42.9)
Adults	26 (92.9)
Older Adults/Elders	5 (17.9)

**Table 6:** Number of people living in the home

Total number of people	Number of Homes (%)
1-2	11 (39.3)
3-4	4 (14.3)
5-6	8 (28.6)
7-8	3 (10.7)
9 or more	2 (7.1)
Total	28

Twenty one percent (6/28) said that they also accessed dental benefits through Social Assistance (Table 7). The majority of participants identified work (46%; 13/28) and/or First Nations and Inuit Health Branch [FNIBH] (68%; 19/28) as sources of dental benefits.

**Table 7:** Source of dental care benefits

Benefit Source	Number of Participants (%)
Social Assistance	6 (21.4)
FNIHB	19 (67.9)
Employment	13 (46.4)
Did Not Answer	2 (7.1)
Total	28

### Knowledge, attitude, and behaviours

The participants had very good knowledge about the impact of healthy baby teeth and children’s teeth to the overall health of children (e.g., baby teeth are very important to the child’s health and development, they help them chew, speak and smile) and described healthy teeth, in general, as having good hygiene and self-care (e.g., flossing, brushing on regular basis), healthy nutrition (e.g., eating healthy, sugar intake is down), or reflecting on the health of teeth (e.g., no cavities or any disease) or appearance of teeth (e.g., healthy teeth means straight teeth). Almost all participants agreed that the health of teeth effect the way children talk, their hearing, their self-confidence and self-esteem, their appearance (e.g., they won’t smile if they have yellow, rotting teeth or have broken teeth) and or the pain they experience.

Participants described both nutrition choices and dental hygiene as reasons why children may develop cavities and decay in baby teeth (e.g., bottle, sugar, juice/iced tea, tea, not brushing). Historically, some participants described that children’s teeth were healthier (e.g., back in my day, we hardly or never had candy or junk food. Back then I hardly knew of anyone who had bad teeth). Traditional practices to keep children’s teeth healthy included, more traditional soothers (e.g., dry meat; used to give them bark to chew on; …suck on some fruit, wrapped in a cloth, (leather) hide to help with teething and ‘Indian soup’ broth and… red willow, grinded, wrapped in broad cloth. Babies suck on it to help teething and strengthen teeth). Brushing teeth was identified as important to keep children’s teeth healthy; however, the frequency of how often children have their teeth brush varied (e.g., not regularly, once a day, to 3 times a day, sometimes more). A lack of understanding was identified related to: how long children needed to be supervised when brushing teeth; when dental assessments should be performed; and the role of fluoridation in preventive care. Often the parents viewed the first contact with dentists or therapists as traumatic or painful.

### Community: strengths/supports

The strengths identified included:

- ▪ having a dental clinic within the community (i.e., access to the dentist and/or dental therapist on reserve).
- ▪ quality of care provided at the dental clinic, and
- ▪ incentives offered at the dental clinic and by the program (e.g., awards, toothbrushes, transportation) were also identified.

### Community: barriers/roadblocks (see Fig. 1)

The existing barriers/roadblocks preventing families in the community from receiving care and information about child oral health included: transportation; knowledge of services available; types of information available (inclusive of communication channels used); language; parents’ and community’s lack of interest; and income (e.g., transportation, not knowing what programs are available, lack of information, welfare, budgets, low community involvement). Access and affordability of nutritious food and dental care supplies were also highlighted as preventing parents or caregivers from providing good care for their children’s teeth (e.g., not getting adequate fruits, veggies and maybe having financial difficulties; not enough money or transportation to town; can’t afford toothbrushes, toothpaste, or floss). Several participants mentioned the challenges in the community to access healthy food (I.e., they had to travel to obtain healthier options), as well as the high cost of food (e.g., fruits are more expensive than chips [and that kind of stuff] so lots of people buy the cheaper option. I was raised on meat so my family still eats primarily that; … on-reserve lots of people are on welfare and can’t afford veggies and fruits).

**Fig. 1:**
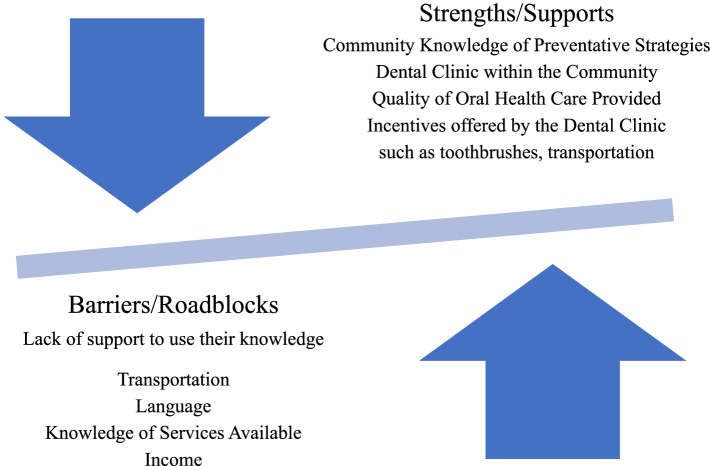
Decrease the Prevalence of ECC

### Community identified ideas for change

The participants shared several thoughts on how to help parents and caregivers take care of young children’s teeth. As stated above, many participants spoke about the importance of access to dental care supplies, education materials to increase awareness and help to address barriers, such as affordability of healthy nutritional foods and dental care services (e.g., more access to floss, brushes, toothpaste, teaching awareness about good practices to parents and kids). Some participants also shared the importance of health workers and programs in the community (e.g., health frontline workers to educate parents; we’re doing what we can in schools). The significant role of family and early interventions was also highlighted (e.g., family members have to start brushing and flossing more; a dental program is huge; proper oral hygiene in family, regular dental visits, and educating them on importance of healthy teeth).

In summary, the ideas included: incentives (e.g., parental incentives such as fruit and veggies so they don’t have to worry about buying them); information about the role of parents/caregivers in teaching and ensuring proper care for their children’s teeth (e.g., show them how bad teeth can get when they’re not taken care of and show them how easy it is to prevent decay); and increasing access to information (e.g., to constantly have available information/gatherings/teaching sessions— on healthy teeth and health eating. Promote it, with constant initiatives).

### Community necessities

Community needs discussed related to oral health care services and prevention programming included transportation, incentives (free dental supplies), and a focus on prevention programs located at the school (e.g., the tooth brushing program in school would help because some kids are rushing to school and could forget to brush their teeth at home). The community expressed a need to expand incentive programs that are offered in schools to include parents and offer free dental supplies and transportation. A need was also shared for an additional dentist or an increase to the range of services the dental therapist provided so community members do not have to travel off reserve (e.g., more options the dentist can do on the reserve instead of sending patients to the city).

Considering these findings, recommendations for improving the oral health of children would also include sharing information about preventive practices related to bottle feeding which was identified as the most frequent form of feeding (41%; 7 of 17). Encouraging age-appropriate methods of cleaning teeth as well as when children should have their first dental visit were also recommended. The dentist or dental therapist were the most frequently mentioned people to best provide information on how to take care of children’s teeth. In addition, the nurse, community health representative, health care provider, teacher and parents were also mentioned.

### Community reflections

Understanding the context of the community is central in developing partnerships and ongoing relationships. Incorporating cultural teachings were significant to the research relationship and research processes. Following proper protocol is critical in authentic engagement.

When entering the research process with communities, it is vital to understand that their time is limited. Community members often fill numerous roles within the community, and research projects are usually in addition to their ongoing workload. Researchers need to be aware of this when timelines are established. When communities experience celebrations or a death, the research processes stop to observe the cultural protocol.

Trust is developed by following the principles of TCPS 2 and respecting cultural practices identified which have been broadly discussed in this manuscript [29]. Developing resources that include trauma-informed care are foundational in supporting the wellbeing of Indigenous peoples [36]. Consideration of languages used in the community and authentic engagement with the community members are necessary in all aspects of the research process.

## Discussion/summary

The participants identified strengths/supports, barriers/roadblocks and solutions to oral health issues in their community. The participants in Thunderchild First Nation have good knowledge about the impact of good oral health on speech, self-confidence, nutrition, and prevention of oral pain. The participants were aware of the preventative strategies such as limiting the use of bottles in duration and contents. There was recognition of the need to emphasize tooth brushing and how to access dental supplies. They also appreciated the attempts by the Community Administrators to have more support for dental services close to the local community including those of a dental therapist. Health Canada has supported the use of dental therapists in communities since the 1970’s [37]. A review of dental therapists, in 50 communities, reported that they expand access to dental care safely and effectively [37].

In this study, the participants identified the difficulty in applying good preventive strategies such as limiting or not using bottles with pressures in crowded homes to quiet and comfort fussing children. There was no discussion of alternative strategies to deal with this circumstance. The participants also noted that the challenge that transportation brings also affects healthy food choices and access to dental services. Holve et al. [38] identified access to healthy food as a necessary community based initiative and they also identify community-based participatory research with Indigenous communities a necessity to guide prevention of ECC.

Suggestions by the participants included that the focus of education should be on prevention that would enhance oral health outcomes in children along with access to dental supplies and reminders of oral health strategies. Community outreach programs focusing on prenatal nutrition have been shown to improve caregivers’ knowledge of ECC and its effects [39, 40]. In 16 Cree communities in Ontario, intervening with expectant mothers to educate about dental care was explored to reduce the prevalence of ECC in infants, but more needs to be done to decrease caregiver strain [40, 41]. The participants also suggested developing more dental supports closer to the community would mitigate the need for transportation and the pressures of access, which is supported by the American Academy of Pediatrics [38]. Lack of access to dental care contributes to oral health disparities [36, 40]. Moreover, strategies for the prevention of ECC need to be co-created with the communities [38, 39].

### Limitations

This research focused on pediatric oral health and was strength-based. Parental experiences with oral health care were not included so the history of oral care in individual families could not be used to help inform the results/findings. Hiring and training community Research Assistants, as well as conducting interviews in person to obtain the survey results, were time and resource intensive, but essential for the outcomes in this research.

Given the nature of the work, the methods and processes can be transferred but the results/findings are not generalizable.

### Knowledge translation

Community-based research and integrated knowledge translation fosters processes that co-create knowledge. This community identified specific knowledge translation processes, including the need for public service announcements in English and Cree over the community radio station and developing key targeted meaningful messages. Similar techniques have been used in Northern Ontario with Indigenous communities consisting of bi-annual media campaigns, pamphlets, and posters in public areas promoting oral health [40].

When working with the community, it would be helpful to link oral health to overall health and wellness, informing youth and parents of resources, and communicating to all levels of the community.

In summary, an approach that invites the community to participate is paramount, as well as timing and teamwork. A book (in progress), translated into Plains Cree, is being co-created with the community to incorporate strengths/supports and highlight the barriers/roadblocks that need to be addressed to improve the oral health of children.

## Conclusions

This research was one of the first to explore oral health requisites with an independent Indigenous community. The study specifically explored the strengths/supports and barriers/roadblocks related to the availability, accessibility, accommodation, affordability, and acceptability of oral health care services. In addition, it also included the beliefs and health behaviours related to oral health and the impact that oral health has on the overall well-being of an individual. Developing authentic community engagement is critical to better understand, identify strengths/supports and barriers/roadblocks to oral health care services, and subsequently to establish new resources with Indigenous communities. Engaging individuals, families, community leaders, health care practitioners, educators, and policy makers will aid in the co-creation of a framework to be used in and with the community. The knowledge gleaned by this interdisciplinary, community–based participatory research team has the potential to build capacity with individuals, communities, provinces, and at the national level. Community-based participatory research used in this project established processes to ensure that any change in and with the community is sustainable and has the potential to prevent dental caries among young children. The research processes increased skills and capacities of the research team and the community resulting in sustainable relationships and research results/findings that will inform clinical practice guidelines (CPGs), policy and future research in this vital area of health and well-being.

## Data Availability

The datasets used and/or analysed during the current study are not available to the public because the data belongs to the community [29]. Data may be available through the corresponding author and negotiation with the community on reasonable request.

## Abbreviations

MS: Mutans streptococci
SDHSP: The Saskatchewan Dental Health Screening Program
ECC: Early Childhood Caries
FNIHB: First Nations and Inuit Health Branch
COHS: Canadian Oral Health Strategy
COHI: Children’s Oral Health Initiative
TCPS: Tri-Council Policy Statement
CHMS: Canadian Health Measures Survey
SPSS: Statistical Package for the Social Sciences
